# A Deep Learning Method Using Gender-Specific Features for Emotion Recognition

**DOI:** 10.3390/s23031355

**Published:** 2023-01-25

**Authors:** Li-Min Zhang, Yang Li, Yue-Ting Zhang, Giap Weng Ng, Yu-Beng Leau, Hao Yan

**Affiliations:** 1 Key Laboratory for Artificial Intelligence and Cognitive Neuroscience of Language, Xi’an International Studies University, Xi’an 610116, China; 2 Faculty of Computing and Informatics, Universiti Malaysia Sabah, Sabah 88400, Malaysia

**Keywords:** speech emotion recognition, gender classification, CNN, BiLSTM

## Abstract

Speech reflects people’s mental state and using a microphone sensor is a potential method for human–computer interaction. Speech recognition using this sensor is conducive to the diagnosis of mental illnesses. The gender difference of speakers affects the process of speech emotion recognition based on specific acoustic features, resulting in the decline of emotion recognition accuracy. Therefore, we believe that the accuracy of speech emotion recognition can be effectively improved by selecting different features of speech for emotion recognition based on the speech representations of different genders. In this paper, we propose a speech emotion recognition method based on gender classification. First, we use MLP to classify the original speech by gender. Second, based on the different acoustic features of male and female speech, we analyze the influence weights of multiple speech emotion features in male and female speech, and establish the optimal feature sets for male and female emotion recognition, respectively. Finally, we train and test CNN and BiLSTM, respectively, by using the male and the female speech emotion feature sets. The results show that the proposed emotion recognition models have an advantage in terms of average recognition accuracy compared with gender-mixed recognition models.

## 1. Introduction

Speech emotion recognition is a computer simulation of the human emotion perception and understanding process. It extracts the acoustic features of emotion from the collected speech signals using a microphone sensor and identifies the mapping relationship between these acoustic features and human emotion. Speech emotion recognition is widely used in the field of human–computer interaction [1,2,3,4]. In the field of medicine, the effective recognition of emotion in speech can be used to improve the intelligibility of speech for people with speech disabilities and help listeners better understand the speech information expressed by the speaker [5]. In the field of education, students who learn online should be analyzed to identify their emotional states and improve the quality of teaching [6]. In the field of criminal investigation, the automatic recognition of speech helps to discover the real emotional state of criminal suspects and their attempt to hide their true emotions, thus assisting in lie detection [7]. Currently, due to the impact of COVID-19, about 15.5% of the global population suffers from some kind of mental illness [8], and speech emotion recognition systems are gradually being applied to the field of mental health counseling. The research on the automatic recognition of speech emotion not only promotes the development of computer technology, but also provides an efficient diagnosis of mental illnesses. Through the early detection, intervention and treatment of mental illness, people’s quality of life can be improved.

In recent years, a lot of work has been carried out to automatically recognize emotional information in speech [9,10,11,12], but the lack of significant improvement in recognition accuracy is still a major problem in the field of speech emotion recognition. Researchers have attempted to explore the method to improve the accuracy of emotion recognition from different perspectives. In the work developed in [10], N. Prombut et al. proposed a speech emotion recognition model for Thai subjects. Mel spectrogram and mel-frequency cepstrum coefficient (MFCC) are used for feature extraction, and emotions are classified by combining a one-dimensional convolutional neural network (Conv1D) and two-dimensional convolutional neural network (Conv2D). This study utilizes a dataset from the VISTEC-depa AI Research Institute of Thailand, which includes 21,562 sound samples. The results show that Conv2D with MFCC achieves the highest accuracy rate of 80.59%. In [13], S. Mirsamadi et al. proposed to automatically distinguish emotion-related speech features using deep learning methods. The authors combine bidirectional LSTMs with a novel pooling strategy. This strategy uses an attention mechanism that enables the network to focus on parts of sentences with high emotional salience. The experiments were carried out on the IEMOCAP corpus, and the highest recognition rate reached 63.5%. In the study of Kwon et al. [14], a lightweight deep learning-based self-attention module (SAM) for a SER system is proposed to address the fact that a low recognition rate and high computational cost result in a scarceness of datasets, model configuration, and pattern recognition. The proposed method shows consistent improvements in experiments for the IEMOCAP, RAVDESS, and EMO-DB datasets, and shows 78.01%, 80.00%, and 93.00% accuracy, respectively. Most of the abovementioned studies aim to improve the accuracy of emotion recognition by applying feature extraction, using model recognition or adding corpus, but they do not obtain a satisfactory result.

At present, acoustic emotional features are widely used to represent emotional information, including rhythm features, quality features, and spectral features. Prosodic features, also known as super segmental features [15], are phonetic features that can be perceived by humans, such as intonation, pitch, sound length, and rhythm. Among the most widely used prosodic features are fundamental frequency, speech energy, and duration. T. Iliou et al. proved that prosodic features can distinguish emotions with different arousal well, but cannot distinguish emotions with the same arousal or valence well [16]. The spectral feature describes the correlation between the shape change of the vocal tract and the vocal movement, and it reflects the short-term spectral characteristics of the signal. Spectral features are obtained by transforming the time–domain signal into a frequency–domain signal using Fourier transform. The most commonly used spectral feature is MFCC. A previous study proposed a new auditory-based spectral feature, which is used for dimensional emotion recognition to obtain temporal dynamic information [17]. The experiment shows that better performance is achieved on the dimension of valence and arousal. Quality features measure the purity, clarity, and intelligibility of speech. This mainly includes bandwidth [18], formant frequency [19], amplitude perturbation [20], etc. Another study utilized sound quality features, such as formants and harmonic-to-noise ratio, distributed in different frequency bands, to conclude that voice features are more suitable for distinguishing emotions with the same arousal and different valence [21].

Emotional expression is based on the acoustic characteristics of the speaker, and these acoustic characteristics are highly influenced by the speaker’s gender. I Bisio et al. proposed a speech emotion recognition algorithm combined with a gender classifier [22]. It builds a gender recognition algorithm using fundamental frequency features, aiming to provide prior information about the speaker’s gender. Further, it uses a support vector machine as a classifier with gender information as input. Experiments on the EMODB dataset achieve a recognition rate of 81.5%. Anish Nediyanchath et al. proposed a multi-head attention deep learning network for speech emotion recognition (SER) based on log mel-filterbank energies (LFBE) spectral features [23]. In addition to multi-head attention and position embedding, multi-task learning with gender recognition as an auxiliary task is applied. The experiments are conducted on the IEMOCAP dataset, and an overall accuracy of 76.4% is achieved. In [24], the authors propose a new emotion recognition algorithm that does not rely on any acoustic features and combines a residual convolutional neural network (R-CNN) with a gender information block. Utilizing the deep learning algorithm, the network automatically selects important information from the original speech signal for the classification layer to complete the emotion recognition. The results show that the proposed algorithm achieves recognition rates of 84.6%, 90.3%, and 71.5% on the CASIA, EMODB, and IEMOCAP datasets, respectively. Although the above studies consider the influence of gender on speech emotion recognition, they only input gender as a characteristic parameter, or manually distinguish different genders and then input the recognition model. The recognition results do not improve significantly.

In this study, based on the differences in physiological and acoustic characteristic parameters between genders, front-end and back-end models are designed to automatically complete gender classification and speech emotion recognition. Our main contributions are summarized in three phases:

First, the front-end model uses MFCC mean and spectrum contrast to extract the features from the original speech, and automatically distinguishes male and female speech through a multilayer perceptron neural network (MLP).

Second, the acoustic speech emotion features are traversed, and one feature parameter is extracted each time. The recognition results are compared using the support vector machine (SVM) to analyze the weight difference of various speech features in the speech recognition of different genders.

Finally, combinations of the feature parameters of different genders are, respectively, input into a convolutional neural network (CNN) and bidirectional long short-term memory (BiLSTM) speech emotion recognition models established in the back-end to realize the emotion recognition of different genders and improve its accuracy.

The following parts of this paper are organized into five sections. In Section 2, the differences in the physiological and acoustic characteristics between male and female voices are introduced. Section 3 presents related research work, gender classification methods, the extraction of the speech feature parameters of different genders, and the structure of the speech emotion recognition models. Section 4 presents the experimental design and experimental results. Section 5 presents the discussion of the proposed technique. Finally, the summarization of the study is presented in Section 6**.**

## 2. Background

The main problem of speech emotion recognition is that the acoustic performance of speech signals is affected not only by emotional factors, but also by many other factors. Among them, the discrepancy of acoustic characteristics caused by the difference in the physiological characteristics of speakers is the main influencing factor that reduces the accuracy of speech emotion recognition.

The vocal tract is the main organ that produces the voice. Its core function is to adjust the timbre of the sound produced by the vibration of the vocal cords. Speech is produced according to the shape of the vocal tract, which changes over time. The shape of the vocal tract depends on the shape or size of the vocal organ, and different genders inevitably show individual differences. Previous studies show that the ratio of the total length of the vocal tract of adult females to that of adult males is about 0.87 [25]. Males and females differ in the thickness of the larynx, angle of the thyroid tablet and shape of the glottis [26]. The vocal cords are part of the vocal tract. The tension or relaxation of the vocal cords determines the pitch of the voice. People of different genders have different vocal cord structures; thus, the pitch of the voice is also different. Adult male and female vocal cords are different in length, thickness, tension, and other anatomical structures, as well as in the physiological functions of respiratory organs and resonance organs, resulting in different characteristics between adult males and females in vocalization and voice quality. The main factor affecting the rate of vocal cord vibration is the length of the vocal cord, and long vocal cords vibrate more slowly than short ones. Previous studies show that the ratio of female vocal cord length to male vocal cord length is about 0.8 [27]; therefore, men’s voices are lower than women’s in most cases. In addition, female voices have a higher base frequency or pitch in comparison with males’. The average formant frequency of female speakers is higher than that of male speakers, and the slope of the female spectrum is steeper than that of male speakers.

In conclusion, there are general physiological differences between genders; thus, men and women may express the same emotion in completely different ways. Therefore, it is necessary to classify emotions according to gender and carry out emotion recognition according to the different acoustic characteristics of males and females to improve the overall accuracy of the recognition system.

## 3. Methods

**Figure 1:**
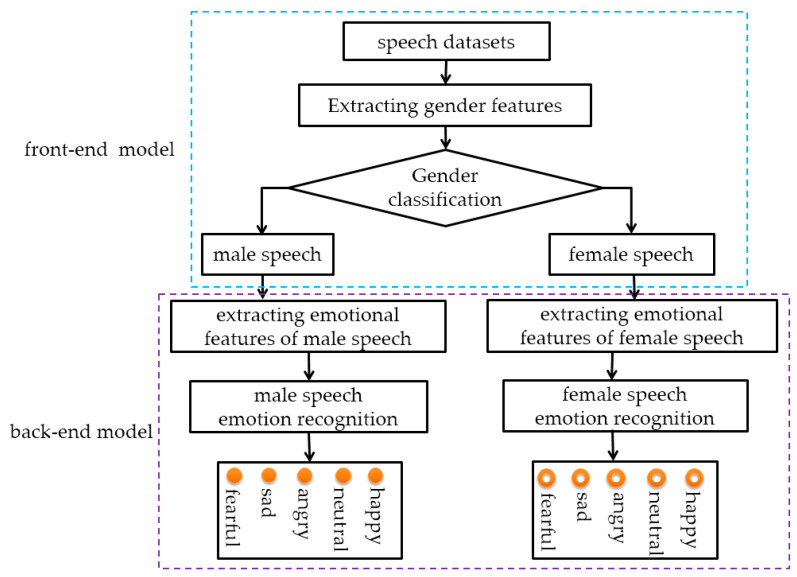
Framework of speech emotion recognition.

In this section, we establish the overall framework of speech emotion recognition. The overall framework shown in Figure 1 is divided into two models: the front-end model and the back-end model. The front-end model completes gender recognition and the classification of speech data. The back-end model extracts emotional features from the speech data of males and females, respectively, and provides the emotional recognition results of different genders.

### 3.1. Gender Recognition

#### 3.1.1. Extraction of Speech Feature Parameters

MFCC is based on the characteristics of the human ear, cochlea, and basement membrane, and has a nonlinear corresponding relationship with the actual frequency, which makes its cepstrum more similar to the nonlinear human auditory system. Previous studies show that MFCC and F0 classify gender well [28]. Spectrum contrast takes into account the peak value, valley value, and the difference value of each sub-frequency spectrum to show the relative characteristics of the spectrum. The spectrum contrast feature can roughly reflect the corresponding distribution of the middle and morning harmonics in the spectrum, retain more spectrum characteristic information, and better distinguish different kinds of speech information. Therefore, MFCC mean, fundamental frequency F0, and spectral contrast ratio are adopted in this study to classify the speech features of different genders more accurately.

#### 3.1.2. Gender Recognition Model

**Figure 2:**
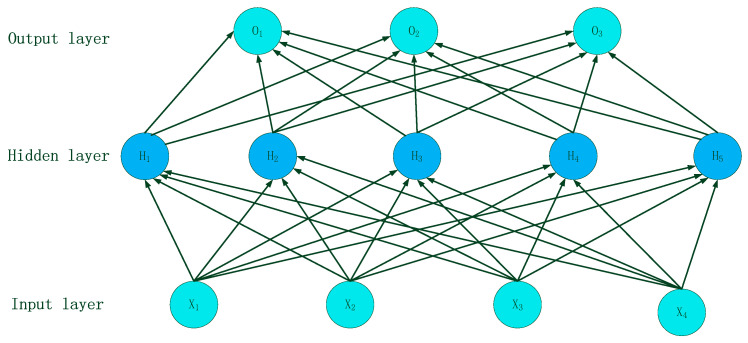
Structure of MLP.

In this study, MLP is used for the automatic gender recognition of detected speech. MLP consists of an input layer, several hidden layers in the middle, and an output layer [29]. It has high parallelism, high nonlinear global function, and good fault tolerance and associative memory function. The structure of MLP is shown in Figure 2.

Let sample $\;X\in R^{n\times d}$, where n denotes the batch size, and d represents the number of inputs. Suppose the multilayer perceptron has just one hidden layer, where the number of hidden units is h, and the output of the hidden layer is $H\in R^{n\times h}$. Since the hidden layer and the output layer are fully connected layers, the weight parameters and deviation parameters of the hidden layer can be set as:

(1) $$W_{h}\in R^{d\times h},\;b_{h}\in R^{1\times h}$$

The weight parameters and deviation parameters of the output layer are:

(2) $$W_{o}\in R^{h\times q},\;b_{o}\in R^{1\times q}$$

Then, the output $O\in R^{n\times q}\;$*of* a multilayer perceptron design with a single hidden layer is calculated as follows:

(3) $$\;H=XW_{h}+b_{h},\;O=HW_{o}+b_{o}$$

The output of the hidden layer is directly taken as the input of the output layer, and the subsequent equation can be obtained:

(4) $$O=\left(XW_{h}+b_{h}\right)W_{o}+b_{o}=XW_{h}W_{o}+b_{h}W_{o}+b_{o}$$

In this study, MFCC mean, fundamental frequency F0, and spectral contrast are used to extract the emotional features from the speech of different genders.

### 3.2. Speech Emotion Recognition

#### 3.2.1. Extraction of Speech Emotion Feature Parameters

For all speech emotion recognition systems, a key problem lies in how to select the best feature set to represent speech emotion signals. This section explores the differences in speech emotion features among speakers of different genders. The rhythm features, quality features, and spectral features are traversed. One feature parameter is extracted each time and input into the SVM model for individual emotion recognition. The recognition results are used to judge the weight of different feature parameters in male and female speech emotion features. The ranking of male and female speech emotion feature parameters is shown in Table 1 (See Appendix A for details). According to the proportion of weight, the feature parameters with a recognition rate greater than 50% are displayed in order from high to low.

As can be seen from Table 1, the weight of male and female speech emotion feature parameters in emotion recognition is significantly different: the male single feature parameter has the best MFCC effect in emotion recognition, followed by its maximum and mean value. On the contrary, women have the best maximum MFCC, followed by the mean and MFCC. For males, sound pressure level (SPL) is more important, followed by voice energy. In the case of female voices, voice energy is more important, followed by Mayer spectrum.

The emotional characteristics of male speech include shimmer abs (absolute amplitude perturbation) and SPL, both of which are not found in females. Female voice emotion feature parameters include zero-crossing rate (ZCR) and short-time energy, while male speech does not. Amplitude perturbation describes the variation of acoustic amplitude between adjacent periods, which mainly reflects hoarseness. The male vocal tract is rougher and hoarser than the female vocal tract; thus, the amplitude perturbation parameters have a greater effect on the male voice. SPL is defined as the root mean square value of the instantaneous pressure generated by a sound wave at a point. In general, women’s vocalizations have more high-frequency components and men’s vocalizations have more low-frequency components. Women speak louder than men at the same SPL. SPL fluctuates more in men and less in women during mood swings. ZCR refers to the number of times a speech signal passes through the zero point (from positive to negative or from negative to positive) in each frame. The ZCR of unvoiced sounds is the highest, while that of voiced sounds is the lowest, and the short-time energy can distinguish between unvoiced and voiced sounds. Voiced sounds are produced by the vibration of vocal cords and contain most of the energy in the sound. It shows obvious periodicity. Female vocal cords are shorter than male vocal cords; therefore, female voices are crisper and male voices are deeper.

#### 3.2.2. Speech Emotion Recognition Models

The gender-based emotion detection method, which incorporates gender information into the process of emotion recognition, has proved to be robust and successful. Studies have shown that gender-mixed emotion recognition systems have a lower success rate than gender-specific systems [24]. To further exclude the possible influence of classifiers on emotion recognition results, CNN and BiLSTM emotion recognition models are established in this study.

CNN and BiLSTM are representative algorithms of deep learning, which have achieved great success in natural speech processing, such as speech recognition and language translation, and have shown excellent performance in speech emotion recognition. In this experiment, these two classifiers are used for a comparative analysis.

In [30], the authors proposed an improved CNN model that greatly enhance CNNs’ capability of modeling geometric transformations. CNN’s model formula is as follows:

(5) $$Z\left(i,j\right)= [Z^{l}w]\left(i,j\right)+b=\overset{K_{l}}{\underset{k=1}{\sum }}\overset{f}{\underset{x=1}{\sum }}\overset{f}{\underset{y=1}{\sum }} [Z_{k}^{l}\left(s_{0}i+x,s_{0}j+y\right)w_{k}^{l+1}\left(x,y\right)]+b\;\left(i,j\right)\in \left\{0,1,\cdots L_{l+1}\right\}\;L_{l+1}=\frac{L_{l}+2p-f}{S_{0}}+1$$

where b is the offset; $Z^{l}\;$ and $\;Z^{l+1}\;$ represent the convolution input and output of the l+1 layer, which are known as the feature graph; $L_{l+1}$ denotes the size of $\;Z^{l+1}$; $Z\left(i,j\right)$ corresponds to the feature graph; $w_{k}^{l+1}\left(x,y\right)\;$ represents the $k_{th}$ sub-convolution kernel matrix of the convolution kernel; k is the number of channels of the feature graph; f is the convolution kernel size; $s_{0}$ is the convolution step size; and *p* is the number of filling layers.

**Figure 3:**
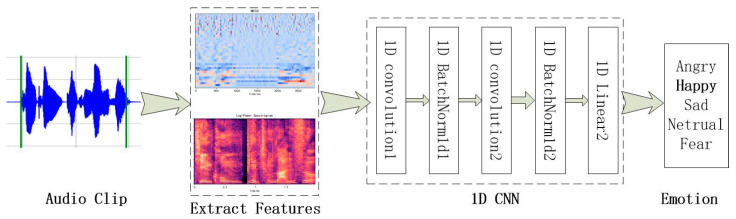
Structure of CNN.

CNN is composed of two convolution layers and a fully connected layer. The window length of the convolution kernel is 5, the convolution step is 1, and the activation function is “Relu”. For each convolution layer, the output of convolution is transferred to one dimension for batch normalization, and the prediction results are obtained after the softmax activation layer. Batch normalization reduces the internal covariance drift in the feature graph by normalizing the output of the previous layer. The irregular effect can reduce the overfitting. The structure of CNN is shown in Figure 3.

The optimizer of CNN selects “Adam”, and the loss function is cross-entropy. In order to prevent overfitting, input neurons are randomly disconnected with a probability of 0.3 every time the parameters are changed during training. In the process of training and testing, five-fold cross-validation is adopted, and 80% of the dataset is used as training data and 20% as test data.

**Figure 4:**
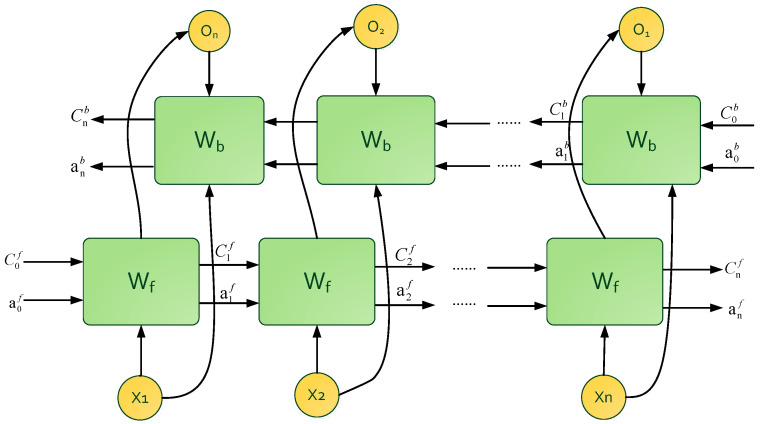
Structure of BiLSTM.

BiLSTM uses a layer of bidirectional LSTM to extract the features of the hidden layer, and directly selects the 256-dimensional feature outputs of the hidden layer for batch normalization [31]. By normalizing the output of the previous layer, the internal covariance drift in the feature graph can be reduced, and the regularization effect caused can reduce the overfitting. Then, a full connection layer is used to down sample the input features and reduce the dimension of feature space. Finally, the prediction results are obtained after the softmax activation layer. The optimizer selects “Adam”. The fixed learning rate is set to 0.001, and the loss function is cross-entropy. In order to prevent overfitting, the neurons are randomly disconnected with a probability of 0.5 every time the parameters are changed in the training process. In the process of training and testing, five-fold cross-validation is adopted, with 80% of the dataset used as training data and 20% as test data. The structure of BiLSTM is shown in Figure 4.

## 4. Experiment Results

### 4.1. Emotional Speech Dataset

In this paper, the Ryerson Audio–Visual Database of Emotional Speech and Song (RAVDESS) and the CASIA Chinese emotional speech dataset are used for testing. The diversity of the data prevents the model from being applicable to only one dataset; therefore, the effect of the model can be fully validated. In the process of training and testing, five-fold cross-validation is adopted, 80% of the dataset is used as training data and 20% as test data.

The RAVDESS is a multimodal dataset, which consists of 24 young and middle-aged actors (12 males and 12 females) uttering sentences that have matching words in neutral North American accents [32]. According to the analysis of speech features and the requirement of emotion recognition, the experiment selected 1440 audio recordings of emotional speech as test samples which contain eight kinds of emotion: happiness, sadness, anger, fear, calm, disgust, surprise, and neutral. The sampling rate of voice data audio is 48 kHz, 16 bit, and the file is saved in an uncompressed waveform format. A total of 1440 experimental samples, including 720 males and 720 females, are used without gender classification.

The CASIA Chinese emotional speech dataset, released by the Institute of Chinese Academy of Sciences, is composed of 1200 sentences vocalized by four young and middle-aged professional orthoepists (two males and two females). Our research chooses six emotions: happiness, sadness, anger, fear, surprise, and neutral. The sampling rate of voice data audio is 16 KHz, 16 bit, and the file is saved in an uncompressed waveform format. A total of 1200 test samples are used, which consist of 600 samples of men and 600 samples of women. See Table 2 for details.

The experiment is carried out on Windows 7, where the computer hardware is configured as Intel i7 CPU at 2.80 GHz, with 16 GB of memory. The programming language is Python 3.8. The running process of the program mainly relies on the CPU for calculation. GPU is not used. The run-time is about 10 min.

### 4.2. Gender Classification Results

The front-end model of this experiment automatically classifies the original speech according to gender. MFCC mean and spectral contrast are used to extract gender features, and the speech gender classification is completed through MLP. Through the test, the accuracy of gender classification in the RAVDESS dataset reaches 100%, while the accuracy in the CASIA dataset is 99.5%.

### 4.3. Speech Emotion Recognition Results

The back-end model of this experiment automatically completes the speech emotion recognition process according to different genders. First, the male and female voices input from the RAVDESS dataset are preprocessed. Acoustic speech feature parameters are extracted and integrated into overall feature vectors according to their respective dimensions which are, respectively, sent into CNN and BiLSTM for speech emotion recognition. The comprehensive recognition result in Table 3 is used to obtain the comprehensive recognition rate of the whole dataset by integrating the recognition rate of the identified males and females.

After the RAVDESS dataset is tested, the procedure is repeated in the CASIA dataset. The final recognition results are shown in Table 3.

As can be seen from Table 3, in the RAVDESS dataset, the comprehensive recognition rate of CNN with gender classification is 9.72% higher than that of CNN without gender classification, while that of BiLSTM is increased by 5.20%. In the CASIA dataset, the comprehensive recognition rate of CNN with gender classification is 7.83% higher than that of CNN without gender classification, while BiLSTM improves by 5.41%. The accuracy is higher when CNN and BiLSTM recognize male and female speech, respectively, while recognizing the speech of all genders together shows a lower accuracy. Moreover, the average accuracy of speech recognition of males is significantly better than that of females. The recognition accuracy of CNN for male speech is 1.39% and 5.83% higher than that of female speech in RAVDESS and CASIA, respectively, while that of BiLSTM for male speech is 3.47% and 9.17% higher. This result indicates that the emotional characteristics of male speech are more significant than those of female speech, and are easier to be recognized by models.

We also find that CNN shows a better performance in speech emotion recognition than BiLSTM. This is because CNN mainly focuses on local features and has a high degree of correlation with the maximum and minimum of speech features, pitch, and other parameters. Local features of speech are more obvious than the overall features, which is conducive to CNN, while BiLSTM focuses on timing features, and is relatively less sensitive to local features.

**Figure 5:**
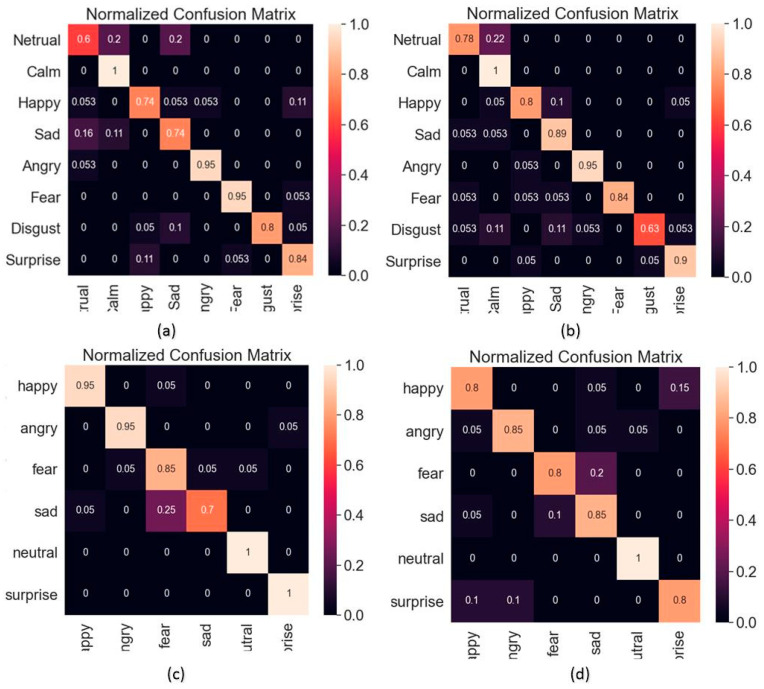
CNN recognition results. (**a**) Result of male speech in RAVDESS dataset. (**b**) Result of female speech in RAVDESS dataset. (**c**) Result of male speech in CASIA dataset. (**d**) Result of female speech in CASIA dataset.

**Figure 6:**
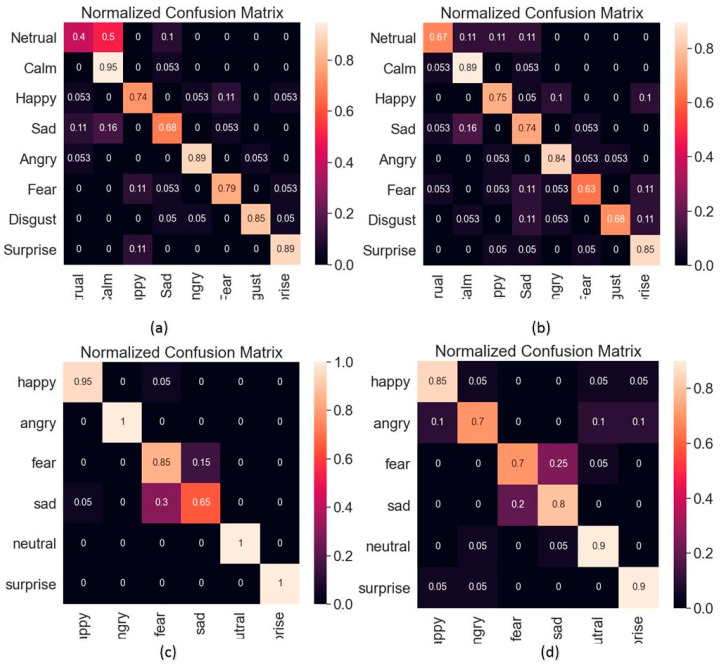
BiLSTM recognition results. (**a**) Result of male speech in RAVDESS dataset. (**b**) Result of female speech in RAVDESS dataset. (**c**) Result of male speech in CASIA dataset. (**d**) Result of female speech in CASIA dataset.

The confusion matrix of CNN recognition results and the confusion matrix of BiLSTM recognition results are shown in Figure 5 and Figure 6, respectively.

## 5. Discussion

Gender-based classification and analysis of the influence weights of multiple speech emotion features in speech emotion recognition across genders are the main contributions of the paper. The MLP model is used to recognize the gender of the original speech and distinguish the male and female speech data. The differences between male and female speech in terms of acoustics are analyzed. We further analyze the influence weights of multiple speech emotion features in male and female speech emotion recognition and establish weight rankings. Then, we build the optimal feature sets for male and female emotion recognition, respectively. Finally, we feed the set of speech emotion features into CNN and BiLSTM models for training and testing. The value of this research approach is to reduce the difficulty of model recognition by differentiating between genders. The establishment of more targeted feature sets will help to further improve the recognition accuracy.

### 5.1. Comparison of Accuracy with Algorithm Based on Deep Learning

Table 4 compares the results of speech emotion recognition in previous studies [24,33,34,35] and this study. Sun, T.W [24] proposed a novel emotion recognition algorithm that does not rely on any speech acoustic features and combines speaker gender information with the emerging R-CNN structure. Kwon, S et al. [33] proposed an artificial intelligence-assisted deep stride convolutional neural network (DSCNN) architecture using the plain nets strategy to learn salient and discriminative features from a spectrogram of speech signals that are enhanced in prior steps to improve performance. Sajjad, M et al. [34] proposed a new strategy for SER by using sequence selections and extraction via a non-linear RBFN-based method to find a similarity level in clustering. Then, they proposed a multilayer deep BiLSTM to learn and recognize long-term sequences in audio data for recognizing emotions. The achieved accuracy for the RAVDESS dataset is 77.02%. None of the abovementioned studies examine the weighting of speech emotion features for different genders, nor do they create separate feature sets for different genders; thus, none of the recognition accuracies are satisfactory.

It can be seen from Table 4 that the method adopted in this study can significantly improve the accuracy of speech emotion recognition, especially in the CASIA dataset, where the results are improved by 3.31% compared with other algorithms.

### 5.2. Comparison of Accuracy with Algorithm Based on Gender Information

Table 5 shows a comparison of the proposed work with [36], in which S. Kanwal et al. describe a feature optimization approach that uses a clustering-based genetic algorithm. They use the gender information as an independent feature map to feed into CNN to train the whole recognition network. The recognition rates of 82.59% for general speakers, 75.49% for male speakers, and 91.12% for female speakers on RAVDESS are obtained in speaker-dependent experiments.

In this study, the influence weight of emotional features of speech in male and female speech emotion recognition is first analyzed. On this basis, the optimal feature sets for male and female emotion recognition are established, respectively, and input into the CNN model and BiLSTM model, respectively. Ultimately, the recognition rate of male speakers is 9.93% higher, the recognition rate of female speakers is 7.09% lower, and the overall recognition rate is 2.13% higher.

Although the proposed algorithm shows excellent recognition performance thus far, it cannot operate in real time. In addition, the computing power of the proposed algorithm is relatively large and cannot be integrated into mobile devices, which also narrows the application scenarios.

In future work, we aim to add more modes, such as images and text, to improve its accuracy. In addition, we intend to train and experiment in more conditions, including age and identification.

## 6. Conclusions

This paper proposes a speech emotion analysis method based on gender recognition. First, gender recognition and automatic gender classification are performed in the speech dataset. Then, based on the different physiological characteristics of males and females, the weight of acoustic emotion characteristic parameters in male and female voice emotions is analyzed. Finally, CNN and BiLSTM are used to establish the speech emotion feature analysis model to recognize the emotion of male and female speech in the RAVDESS and CASIA datasets, respectively. The results show that the speech emotion recognition model based on gender classification proposed in this paper effectively improves the accuracy of speech emotion recognition. Moreover, the proposed model shows better performance in the emotion recognition of male speech compared with female speech.

## Figures and Tables

**Table 1:** Ranking of male and female speech emotion feature parameters.

Ranking	Female Speech Emotion Feature Parameters	Male Speech Emotion Feature Parameters
1	mfccsmax	mfccs
2	mfccsstd	mfccsstd
3	mfccs	mfccsmax
4	energy	sound pressure level
5	mel	energy
6	zero-crossing rate	chroma cens
7	short-time energy	shimmer abs
8	chroma cens	mel

**Table 2:** Emotional speech dataset.

Dataset	Method	Total
RAVDESS	All genders	1440
	Male	720
	Female	720
CASIA	All genders	1200
	Male	600
	Female	600

**Table 3:** Results of speech emotion recognition.

Dataset	Method	CNN			BiLSTM		
		Male	Female	Comprehensive	Male	Female	Comprehensive
RAVDESS	Mixed gender	/	/	75.00%	/	/	72.92%
	Proposed	85.42%	84.03%	84.72%	79.86%	76.39%	78.12%
CASIA	Mixed gender	/	/	80.08%	/	/	80.00%
	Proposed	90.83%	85.00%	87.91%	90.00%	80.83%	85.41%

**Table 4:** Comparison of recognition results between previous works and proposed methods.

Related Works	Dataset	Model	Emotion	Male Recognition Rate	Female Recognition Rate	Comprehensive Recognition Rate
Sun, T.W [24]	CASIA	CNN	Anger, fear, neutral, happiness, sadness, surprise	/	/	84.60%
Kwon, S et al. [33]	RAVDESS	DSCNN	Neutral, calm, sadness, happiness, anger, fear, disgust, surprise	/	/	79.50%
Sajjad, M. et al. [34]	RAVDESS	CNN	Neutral, calm, sadness, happiness, anger, fear, disgust, surprise	/	/	77.02%
Matin, R. et al. [35]	RAVDESS	SVM	Neutral, calm, sadness, happiness, anger, fear, disgust, surprise	/	/	77.00%
Proposed	RAVDESS	CNN	Neutral, calm, sadness, happiness, anger, fear, disgust, surprise	85.42%	84.03%	84.72%
	CASIA	CNN	Anger, fear, neutral, happiness, sadness, surprise	90.83%	85.00%	87.91%

**Table 5:** Comparison of accuracy with algorithm based on gender information.

Related Works	Dataset	Model	Emotion	Male Recognition Rate	Female Recognition Rate	Comprehensive Recognition Rate
Kanwal, S. et al. [36]	RAVDESS	SVM	Neutral, calm, sadness, happiness, anger, fear, disgust, surprise	75.49%	91.12%	82.59%
Proposed	RAVDESS	CNN	Neutral, calm, sadness, happiness, anger, fear, disgust, surprise	85.42%	84.03%	84.72%
	CASIA	CNN	Anger, fear, neutral, happiness, sadness, surprise	90.83%	85.00%	87.91%

## Data Availability

The RAVDESS dataset used in this study can be accessed from “https://doi.org/10.5281/zenodo.1188976”. The CASIA dataset used in this study is available from the author upon request (lm.zhang@xisu.edu.cn).

## Abbreviations

SAM	self-attention module
MFCC	mel-frequency cepstrum coefficient
R-CNN	residual convolutional neural network
MLP	multilayer perceptron neural network
SVM	support vector machine
CNN	convolutional neural network
BiLSTM	bidirectional long short-term memory
SPL	sound pressure level
ZCR	zero-crossing rate
DSCNN	deep stride convolutional neural network

## Appendix A

mel	Mel spectrogram. The speech signal is converted into the corresponding spectrogram, the data on which are utilized as the feature of the signal.
MFCC	Cosine transform is performed after the Mel spectrogram is obtained, and some of the coefficients are taken.
mfccsmax	the maximum value of MFCC.
mfccsstd	the variance of MFCC.
mfccs	the average value of MFCC.
Pitch	the vibration frequency of the vocal cords.
Formants	frequencies produced by physical vibrations of objects that do not change in pitch.
Spectral contrast	the centroid of the spectrum.
Zero-crossing rate	the number of times the speech signal passes through the zero point (from positive to negative or from negative to positive) in each frame.
Energy	the loudness of the sound, also known as volume.
Short-time energy	the sum of the squares of the amplitude values of the frame speech signal.
chroma_cens	the normalization of chromatographic energy, which converts the speech signal into the corresponding spectrogram and performs normalization processing.
Sound pressure level	The pressure level of a sound. Take the common logarithm of the ratio of the sound pressure to be measured p to the reference sound pressure p(ref) and multiply it by 20. The unit is decibels.
Shimmer abs	The absolute value of shimmer. Shimmer describes the change of sound wave amplitude between adjacent periods, mainly reflecting the degree of hoarseness.

## References

[1] Alnuaim A.A., Zakariah M., Alhadlaq A., Shashidhar C., Hatamleh W.A., Tarazi H., Shukla P.K., Ratna R. (2022). Human-Computer Interaction with Detection of Speaker Emotions Using Convolution Neural Networks. Comput. Intell. Neurosci..

[2] Wani T.M., Gunawan T.S., Qadri S.A.A., Kartiwi M., Ambikairajah E. (2021). A Comprehensive Review of Speech Emotion Recognition Systems. IEEE Access.

[3] Karpov A., Yusupov S. (2018). Multimodal interfaces of human–computer interaction. Her. Russ. Acad. Sci..

[4] Ramakrishnan S., Emary S. (2013). Speech emotion recognition approaches in human computer interaction. Telecommun. Syst..

[5] Zisad S.N., Hossain M.S., Andersson K. Speech emotion recognition in neurological disorders using convolutional neural network. Proceedings of the International Conference on Brain Informatics.

[6] Liu J.J., Wu X.F. (2019). Prototype of educational affective arousal evaluation system based on facial and speech emotion recognition. Int. J. Inf. Educ. Technol..

[7] Nasri H., Ouarda W., Alimi A.M. ReLiDSS: Novel lie detection system from speech signal. Proceedings of the AICCSA.

[8] Ritchie H., Roser M. Mental Health. https://ourworldindata.org/mental-health.

[9] Cheng S., Zhang D., Yin D. A DenseNet-GRU technology for Chinese speech emotion recognition. Proceedings of the ICFEICT.

[10] Prombut N., Waijanya S., Promri N. Feature extraction technique based on Conv1D and Conv2D network for Thai speech emotion recognition. Proceedings of the NLPIR.

[11] Niu Y., Zou D., Niu Y., He Z., Tan H. Improvement on speech emotion recognition based on deep convolutional neural networks. Proceedings of the ICCAI.

[12] Marczewski A., Veloso A., Ziviani N. Learning transferable features for speech emotion recognition. Proceedings of the ACM Multimedia.

[13] Mirsamadi S., Barsoum E., Zhang C. Automatic speech emotion recognition using recurrent neural networks with local attention. Proceedings of the ICASSP.

[14] Kwon S. (2021). Att-Net: Enhanced emotion recognition system using lightweight self-attention module. Appl. Soft Comput. J..

[15] Mahdhaoui A., Chetouani M., Zong C. Motherese detection based on segmental and supra-segmental features. Proceedings of the Pattern Recognit.

[16] Iliou T., Anagnostopoulos C.-N. Statistical evaluation of speech features for emotion recognition. Proceedings of the 2009 Fourth International Conference on Digital Telecommunications.

[17] Peng Z., Dang J., Unoki M., Akagi M. (2021). Multi-resolution modulation-filtered cochleagram feature for LSTM-based dimensional emotion recognition from speech. Neural Netw..

[18] Kent R.D., Vorperian H.K. (2018). Static measurements of vowel formant frequencies and bandwidths: A review. J. Commun. Disord..

[19] Kawitzky D., McAllister T. (2020). The effect of formant biofeedback on the feminization of voice in transgender women. J. Voice.

[20] Gelfer M.P., Fendel D.M. (1995). Comparisons of jitter, shimmer, and signal-to-noise ratio from directly digitized versus taped voice samples. J. Voice.

[21] Borchert M., Dusterhoft A. Emotions in speech-experiments with prosody and quality features in speech for use in categorical and dimensional emotion recognition environments. Proceedings of the 2005 International Conference on Natural Language Processing and Knowledge Engineering.

[22] Bisio I., Delfino A., Lavagetto F., Marchese M., Sciarrone A. (2013). Gender-driven emotion recognition through speech signals for ambient intelligence applications. IEEE Trans. Emerg. Top. Comput..

[23] Nediyanchath A., Paramasivam P., Yenigalla P. Multi-head attention for speech emotion recognition with auxiliary learning of gender recognition. Proceedings of the ICASSP.

[24] Sun T.W. (2020). End-to-End Speech Emotion Recognition with Gender Information. IEEE Access.

[25] Fant G. (1976). Vocal tract energy functions and non-uniform scaling. J. Acoust. Soc. Jpn..

[26] Titze I.R. (1987). Physiology of the female larynx. J. Acoust. Soc. Am..

[27] Hirano M. (1983). Growth, development and aging of human vocal fold. Vocal Fold Physiol..

[28] Levitan S.I., Mishra T., Bangalore S. Automatic identification of gender from speech. Proceedings of the Speech Prosody.

[29] Guha D.R., Patra S.K. Cochannel Interference Minimization Using Wilcoxon Multilayer Perceptron Neural Network. Proceedings of the Telecommunication and Computing.

[30] Dai J., Qi H., Xiong Y., Li Y., Zhang G., Hu H., Wei Y. Deformable convolutional networks. Proceedings of the ICCV.

[31] Hochreiter S., Schmidhuber J. (1997). Long short-term memory. Neural Comput..

[32] Livingstone S.R., Russo F.A. (2018). The Ryerson Audio-Visual Database of Emotional Speech and Song (RAVDESS): A dynamic, multimodal set of facial and vocal expressions in North American English. PLoS ONE.

[33] Kwon S. (2019). A CNN-Assisted Enhanced Audio Signal Processing for Speech Emotion Recognition. Sensors.

[34] Sajjad M., Kwon S. (2020). Clustering-Based Speech Emotion Recognition by Incorporating Learned Features and Deep BiLSTM. IEEE Access.

[35] Matin R., Valles D. A speech emotion recognition solution-based on support vector machine for children with autism spectrum disorder to help identify human emotions. Proceedings of the IETC.

[36] Kanwal S., Asghar S. (2021). Speech Emotion Recognition Using Clustering Based GA-Optimized Feature Set. IEEE Access.

